# Denosumab and bone loss in uncemented total hip arthroplasty: a secondary 5-year follow-up of a randomized controlled trial

**DOI:** 10.2340/17453674.2026.45695

**Published:** 2026-04-01

**Authors:** Caroline SKÖLD, Nils P HAILER, Hans MALLMIN

**Affiliations:** Department of Surgical Sciences/Orthopaedics, Uppsala University, Sweden

## Abstract

**Background and purpose:**

Denosumab may preserve periprosthetic bone mineral density (pBMD) around uncemented total hip arthroplasty (THA) components. This exploratory analysis of a previously published randomized controlled trial (RCT) aimed to assess the effects of denosumab on BMD 5 years after treatment cessation.

**Methods:**

64 non-osteoporotic patients undergoing uncemented THA were enrolled in a randomized, double-blind, placebo-controlled phase-2 trial and received either 2 doses of denosumab or placebo. The primary outcome was pBMD at 12 months, measured by dual-energy X-ray absorptiometry (DEXA). At a mean follow-up of 5.6 years (range 4.3–7.3), 54 patients remained for clinical assessment, DEXA, and plain radiography. The study was registered on ClinicalTrials.gov (NCT01630941).

**Results:**

No differences in pBMD in the acetabular Digas zones or femoral Gruen zones were found between the groups at 5 years. The estimated mean difference in the sum of all zones around the cup was 0.042 g/cm² (95% confidence interval [CI] –0.31 to 0.35; P = 0.8), and for the sum of all Gruen zones –0.06 g/cm² (CI –0.55 to 0.43; P = 0.8). No statistically significant differences were observed in patient-reported outcome measures or the incidence of heterotopic ossification. A gradual decline in pBMD was evident.

**Conclusion:**

At 5 years, the adjusted between-group difference and its 95% confidence interval showed no statistically or clinically relevant effect of denosumab. Whether longer treatment duration or a sequential post-denosumab regimen could influence long-term periprosthetic bone preservation is unknown.

Periprosthetic bone loss is a major contributor to aseptic loosening after total hip arthroplasty (THA), particularly in the proximal femur where postoperative remodeling and stress shielding can compromise long-term implant fixation [1]. Pharmacological strategies to counteract early periprosthetic bone loss—most commonly bisphosphonates—have shown only modest and transient effects, with limited or uncertain clinical relevance [2–6]. Importantly, several randomized trials have demonstrated that preservation of periprosthetic bone mineral density (pBMD) does not consistently translate into improved fixation. Thus, preservation of proximal bone does not reduce early migration of uncemented femoral stems [2,7,8]. One of these trials explicitly reported that neither baseline BMD nor changes in BMD predicted early implant stability, stating that “initial BMD and change in BMD do not appear to be significant determinants of implant early stability in a population without pre-existing metabolic bone disease” [2]. Consistent with this, randomized trials of both bisphosphonates and denosumab in cementless THA have failed to demonstrate clinically meaningful reductions in early femoral stem subsidence despite attenuation of proximal bone loss[2,7,9].

Although denosumab has been shown to reduce early implant migration in cemented total knee arthroplasty (TKA) [10], these findings cannot be extrapolated to cementless THA, given the fundamentally different mechanics and biology in the very different settings of cemented TKA and uncemented THA.

Denosumab, a monoclonal antibody targeting RANKL, is a potent antiresorptive agent that increases BMD and reduces fracture risk, with particularly pronounced effects on cortical bone [11,12]. These pharmacological properties generated interest in its potential to counteract periprosthetic bone loss after THA, and short-term studies—including our previous randomized, placebo-controlled trial—have shown that denosumab effectively attenuates early pBMD loss around uncemented stems [9,13,14]. However, this benefit diminishes after treatment cessation, accompanied by an increase in bone turnover markers. Recent systematic reviews and meta-analyses also support the efficacy of denosumab in increasing pBMD, particularly in clinically relevant regions of the proximal femur [15].

Increasing attention has been drawn to the safety profile of antiresorptive therapy in non-osteoporotic patients with an arthroplasty. Registry-based studies have shown an increased risk of periprosthetic fractures in younger (< 65 years), normal-BMD THA patients treated with bisphosphonates [3], with similar findings in TKA [4]. Because bisphosphonates and denosumab share overlapping adverse-effect profiles—including the potential for atypical fractures—and because current practice recommends sequential bisphosphonate therapy after denosumab discontinuation [16–20], these considerations warrant explicit acknowledgment. Importantly, this safety data was not available at the time of the original RCT-study design.

In this exploratory follow-up of a cohort previously included in an RCT, we evaluated whether the early pBMD gains observed during 1 year of denosumab treatment in non-osteoporotic THA patients are maintained after about 5 years.

## Methods

We aimed to assess the medium-term effects of denosumab and to investigate the impact of any rebound phenomenon after treatment cessation in a previously published randomized controlled trial. A prospective, randomized, double-blind, placebo-controlled phase 2 trial was conducted at Uppsala University Hospital between August 2012 and January 2015 to evaluate the efficacy of denosumab in preventing pBMD loss following uncemented THA in patients with unilateral hip osteoarthritis (OA). 64 participants were randomized to receive either 2 subcutaneous doses of denosumab (60 mg, n = 32) or placebo (n = 32), administered 1–3 days postoperatively and again at 6 months. The primary outcome was BMD in Gruen zone 7 and the combined BMD of Gruen zones 1–7 at 12 months and has previously been reported by Nyström et al. [9].

We now report on an exploratory 5-year follow-up that was not pre-specified in the original trial registration or protocol. The mid-term outcomes therefore represent exploratory post-hoc analyses of secondary and tertiary endpoints based on the cohort previously investigated within the framework of our RCT. No additional sample size calculation was performed for the 5-year analyses, and the results should be interpreted in this context.

The study is reported according to the CONSORT guidelines.

### Participants

Inclusion criteria required radiographic evidence of unilateral OA (Kellgren–Lawrence grade 3 or 4). Key exclusion criteria included contralateral OA (grade > 1 or prior THA), obesity (body mass index [BMI] > 35 or bodyweight > 110 kg), use of bone-modulating medications, malignancy, substance abuse, pregnancy, or other significant comorbidities.

All participants initiated calcium (500 mg) and vitamin D_3_ (800 IU) supplementation 7–14 days preoperatively, continued for 12 months, in line with the recommendation for the entire denosumab treatment period but no longer.

The treatment allocation sequence was generated using permuted block randomization with a fixed block size of 4. An independent trial statistician conducted the randomization using the PROC PLAN procedure in SAS version 9.4 (SAS Institute, Cary, NC, USA). Sequential treatment assignments were then placed in sealed, opaque envelopes. Both participants and investigators were blinded to group allocation. The injectables were identical in appearance.

### Intervention and surgical procedure

THA was performed by 1 of 2 experienced orthopedic surgeons using a cementless technique. All participants received a Continuum acetabular cup (Zimmer Biomet, Warsaw, IN, USA) with a highly cross-linked polyethylene liner, a collum femoris-preserving femoral Ti6Al4V stem (Link CFP, Waldemar LINK GmbH & Co. KG, Hamburg, Germany), and a 28-mm cobalt–chromium femoral head. The first subcutaneous injection (denosumab or placebo) was given postoperatively before discharge; the second dose was administered at 6 months. NSAIDs were not administered at all because of their potential bone-modulating effects.

### Variables and outcome measures

**Figure 1:**
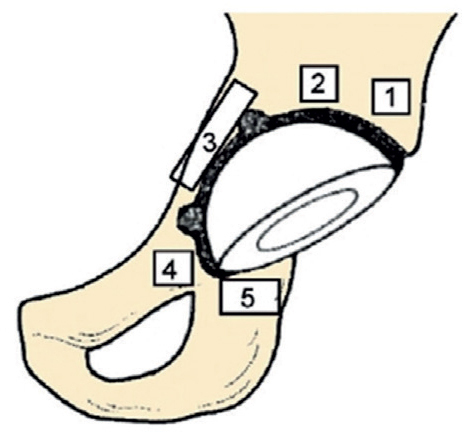
Illustration of the 5 acetabular regions of interest (Digas zones 1–5) used for periprosthetic bone mineral density assessment by DEXA following total hip arthroplasty [21].

**Figure 2:**
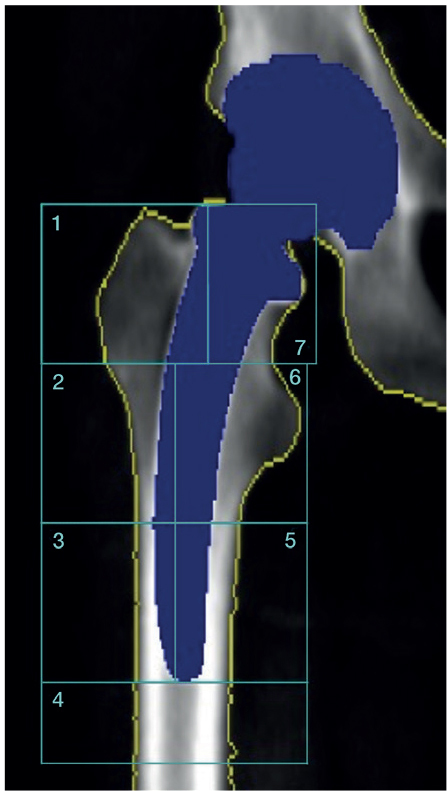
DEXA-based illustration of the 7 Gruen zones used to assess periprosthetic bone mineral density in the proximal femur following uncemented total hip arthroplasty [22].

The outcome of this exploratory follow-up was pBMD at 60 months, assessed in the acetabular (Digas zones) (Figure 1) [21] and femoral (Gruen zones) (Figure 2) [22] regions using dual-energy X-ray absorptiometry (DEXA). Other outcomes included clinical function measured by the Harris Hip Score (HHS) and EQ-5D VAS at all study time points. Implant survival defined as any revision of the stem or cup was recorded.

The participants underwent systematic clinical assessment at 60 months postoperatively. Those reporting back pain were referred for thoracic and lumbar spine radiographs to detect vertebral fractures or degenerative changes.

Heterotopic ossification was evaluated using conventional anteroposterior hip radiographs and graded according to Brooker et al. by a single author (CS) [23].

### Sample size and power considerations

No formal power calculation was performed for this exploratory analysis of a previous RCT; the sample size was based on the original phase 2 trial design [9].

### Statistics

All outcomes were summarized using descriptive statistics, presented as means with standard deviations (SD) or medians with ranges, depending on data distribution.

Between-group comparisons at 60 months were performed using analysis of covariance (ANCOVA). For each continuous outcome, we fitted a linear regression model with the 60-month value as the dependent variable, randomized treatment group as the independent variable of interest, and the corresponding baseline value as a covariate. This approach provides adjusted mean between-group differences and associated 95% confidence intervals (CI) and 2-sided P values.

Implant revision and heterotopic ossification were analyzed as binary variables and summarized using counts and percentages; between-group differences were assessed using chi-square tests or Fisher’s exact tests when expected cell counts were ≤ 5.

Missing data at 60 months was handled using complete case analysis. Given the randomized design, the low number of events, and the observed dropout pattern, the assumption of missing at random (MAR) was considered reasonable. No imputation was performed.

The 5-years analyses were exploratory and not part of the original power calculation; therefore, no adjustment for multiplicity was applied, and P values should be interpreted with caution and in the context of clinical relevance.

All statistical analyses were performed in R version 4.2.2 (R Foundation for Statistical Computing, Vienna, Austria).

### Ethics, registration, data sharing plan, funding, and disclosures

Ethical approval was granted by the Regional Ethics Committee in Uppsala, Sweden (Dnr 2011/297/1–4), with a subsequent amendment approved by the Swedish Ethical Review Authority (Dnr 2023-04551-02). All participants provided written informed consent after receiving both oral and written information about the study.

The trial was registered at ClinicalTrials.gov (identifier: NCT01630941), and a prespecified protocol was in place prior to participant enrolment. Patients were not involved in the design, conduct, or reporting of this research.

No formal data sharing plan has been established. However, the authors are open to reasonable data access requests for academic purposes, subject to approval by the Ethical Review Authority and applicable data protection regulations.

The study was funded by Uppsala University, the Regional Research Council of Uppsala-Örebro, Stiftung Endoprothetik (grant no. S 03/10), and Skobranschens Utvecklingsfond. The funders had no role in study design, data collection, data analysis, data interpretation, or writing of the article. The corresponding author had full access to all data and held the final responsibility for the decision to submit the manuscript.

During the preparation of this work the author used ChatGPT to improve readability and language. After using this tool/service, the authors reviewed and edited the content as needed and take full responsibility for the content of the publication.

Potential conflicts of interest are disclosed in a separate document as required during submission. Complete disclosure of interest forms according to ICMJE are available on the article page, doi: 10.2340/17453674.2026.45695

## Results

### Participant flow and follow-up

**Figure 3:**
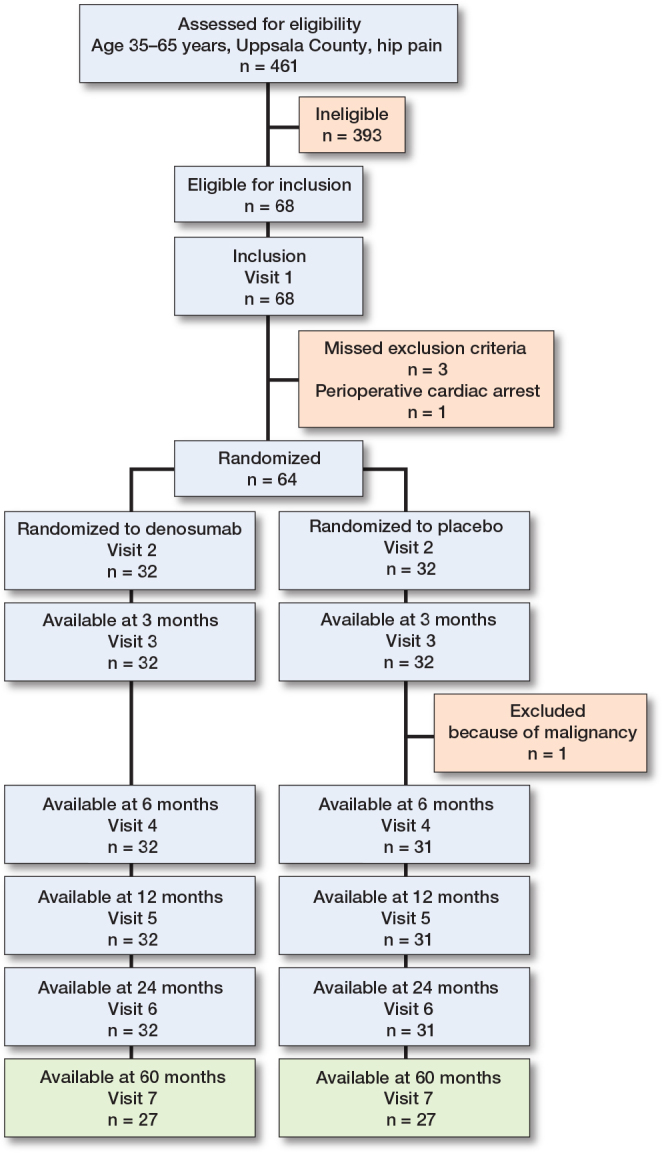
Flowchart for the study.

461 patients aged 35–65 years were screened. 54 participants (84%) were available for clinical and imaging assessment on average 5.6 years (range 4.3–7.3) after the index procedure (Figure 3).

Baseline characteristics of all participants included in the original RCT were similar between the 2 treatment groups (Table 1). No participant fulfilled the World Health Organization (WHO) criteria for osteoporosis, defined as a T-score < –2.5.

**Table 1:** Baseline characteristics. Z-score (age- and sex-matched and weight-adjusted comparison with a white Caucasian US reference population), published to demonstrate that there were no significant baseline differences between the groups. Values are mean (standard deviation) unless otherwise specified

Characteristic	Denosumab (n = 32)	Placebo (n = 32)
Age	58 (5)	59 (5)
Male, n (%)	12 (38)	13 (41)
Body mass index	27 (4)	27 (3)
Harris Hip Score, median (range)	58 (28–81)	51 (33–77)
Z-score, total hip (unaffected) **^a^**	0.58 (1.13)	0.65 (0.67)
Z-score, total hip (affected) **^a^**	0.33 (1.20)	0.33 (0.91)
Z-score, L1–L4 **^a^**	0.91 (1.18)	0.83 (0.91)
T-score, total hip (unaffected)	−0.12 (1.15)	−0.02 (0.71)
T-score, total hip (affected)	−0.38 (1.24)	−0.39 (0.92)
T-score, L1–L4	0.14 (1.22)	0.03 (1.11)
Proportion with osteopenia, n (%) **^b^**		
Total hip (unaffected)	4 (13)	3 (9)
Total hip (affected)	12 (38)	8 (25)
L1–L4	5 (16)	6 (19)

a Age- and sex-matched and weight-adjusted comparison with a white US reference population.

b WHO definition T-score < –1.0 and > –2.5.

### Periprosthetic bone mineral density

**Figure 4:**
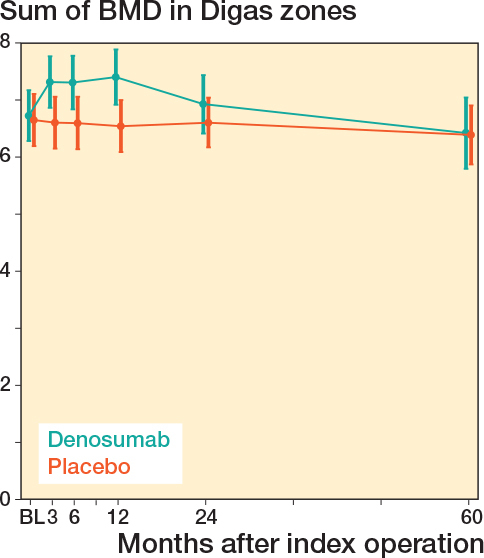
Sum of mean pBMD with 95% confidence interval for all Digas zones around the cup in the acetabulum. No difference was found 5 years after the index procedure. BL = 1–3 days after surgery.

**Figure 5:**
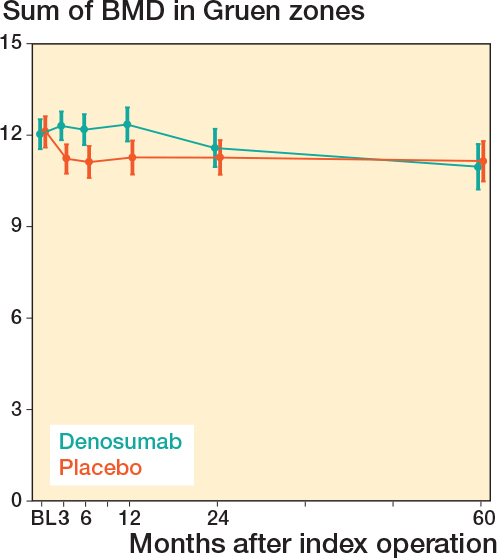
Sum of mean pBMD with 95% confidence interval for all Gruen zones around the stem in the femur. No difference was found 5 years after the index procedure. BL = 1–3 days after surgery.

No statistically significant differences in pBMD were observed between treatment groups at the 60-month follow-up, either in the acetabular (Digas) or femoral (Gruen) regions (Table 2). The estimated difference in the sum of all Digas zones was 0.042 g/cm² (CI –0.31 to 0.35; P = 0.8, Figure 4), and in the sum of all Gruen zones –0.06 g/cm² (CI –0.55 to 0.43; P = 0.8, Figure 5).

**Table 2:** Mean pBMD (g/cm^2^) and (standard deviation) for each acetabular Digas zone and femoral Gruen zone immediately after surgery (0) and at 12, 24 and 60 months, at medium-term follow-up

Outcome	Months after surgery	Denosumab	Placebo	P value ^a^
Digas 1	0	1.93 (0.39)	1.99 (0.36)	
	12	2.12 (0.42)	1.89 (0.39)	
	24	1.91 (0.40)	1.85 (0.38)	
	60	1.73 (0.45)	1.84 (0.37)	> 0.9
Digas 2	0	1.48 (0.36)	1.52 (0.33)	
	12	1.64 (0.36)	1.41 (0.27)	
	24	1.50 (0.33)	1.39 (0.23)	
	60	1.34 (0.38)	1.33 (0.25)	0.5
Digas 3	0	1.47 (0.34)	1.39 (0.35)	
	12	1.59 (0.37)	1.38 (0.39)	
	24	1.48 (0.36)	1.42 (0.37)	
	60	1.38 (0.37)	1.31 (0.40)	> 0.9
Digas 4	0	0.86 (0.51)	0.80 (0.44)	
	12	0.99 (0.54)	0.82 (0.41)	
	24	0.97 (0.54)	0.85 (0.42)	
	60	0.88 (0.51)	0.79 (0.39)	0.6
Digas 5	0	0.99 (0.27)	0.92 (0.28)	
	12	1.07 (0.29)	1.00 (0.25)	
	24	1.07 (0.30)	1.04 (0.22)	
	60	1.10 (0.27)	1.09 (0.28)	0.5
Digas sum	0	6.73 (1.23)	6.66 (1.19)	
	12	7.41 (1.34)	6.55 (1.19)	
	24	6.93 (1.42)	6.61 (1.14)	
	60	6.43 (1.55)	6.40 (1.22)	0.8
Gruen 1	0	0.88 (0.16)	0.89 (0.16)	
	12	0.97 (0.21)	0.87 (0.22)	
	24	0.86 (0.23)	0.88 (0.22)	
	60	0.82 (0.23)	0.89 (0.23)	0.3
Gruen 2	0	1.56 (0.22)	1.52 (0.20)	
	12	1.63 (0.26)	1.41 (0.24)	
	24	1.47 (0.28)	1.38 (0.23)	
	60	1.30 (0.31)	1.30 (0.22)	0.4
Gruen 3	0	2.23 (0.29)	2.25 (0.27)	
	12	2.29 (0.30)	2.17 (0.25)	
	24	2.27 (0.29)	2.19 (0.25)	
	60	2.26 (0.31)	2.21 (0.27)	0.2
Gruen 4	0	2.14 (0.30)	2.11 (0.28)	
	12	2.15 (0.32)	2.03 (0.29)	
	24	2.08 (0.33)	2.02 (0.29)	
	60	2.01 (0.37)	2.03 (0.31)	0.4
Gruen 5	0	2.20 (0.29)	2.22 (0.28)	
	12	2.21 (0.30)	2.11 (0.26)	
	24	2.16 (0.30)	2.15 (0.26)	
	60	2.14 (0.28)	2.17 (0.27)	0.8
Gruen 6	0	1.48 (0.21)	1.56 (0.24)	
	12	1.56 (0.26)	1.46 (0.27)	
	24	1.44 (0.30)	1.43 (0.24)	
	60	1.33 (0.30)	1.42 (0.27)	0.4
Gruen 7	0	1.52 (0.18)	1.57 (0.23)	
	12	1.54 (0.23)	1.21 (0.25)	
	24	1.30 (0.28)	1.21 (0.31)	
	60	1.11 (0.32)	1.12 (0.30)	0.6
Gruen sum	0	12.02 (1.37)	12.11 (1.40)	
	12	12.35 (1.55)	11.26 (1.46)	
	24	11.58 (1.72)	11.27 (1.49)	
	60	10.96 (1.84)	11.13 (1.61)	0.8
Total hip BMD (unaffected)	0	1.03 (0.16)	1.04 (0.11)	
	12	1.04 (0.16)	1.04 (0.11)	
	24	1.03 (0.16)	1.04 (0.11)	
	60	1.04 (0.18)	1.03 (0.12)	0.3
L1–L4 BMD	0	1.23 (0.16)	1.21 (0.15)	
	12	1.27 (0.17)	1.21 (0.15)	
	24	1.23 (0.16)	1.22 (0.15)	
	60	1.27 (0.18)	1.24 (0.15)	0.7

Results from 0, 12, and 24 months have been published previously for Digas and Gruen zones but not for unaffected hip or L1–L4 [9,13].

a P value for treatment at 60 months from ANCOVA model adjusted for baseline values.

### Clinical outcome

Harris Hip Score and EQ-5D VAS were similar between groups at all time points, and no statistically significant differences were detected (Table 3).

**Table 3:** Harris Hip Score (HHS) and patient-reported pain according to Visual Analogue Scale (VAS). Values are mean (standard deviation)

Outcome	Months after surgery	Denosumab	Placebo	P value
HHS	0	55 (12)	51(11)	
	12	91 (9.2)	94 (10)	
	24	95 (6.9)	96 (7.7)	
	60	96 (7.5)	95 (7.7)	0.7
VAS	0	50 (22)	51 (23)	
	12	84 (10)	87 (14)	
	24	88 (11)	90 (13)	
	60	87 (8.7)	82 (17)	0.3

Results from 0, 12, and 24 months have been published previously [9,13].

### Back pain and vertebral compression

5 participants reported back pain at the follow-up visit after 60 months, 3 in the placebo group and 2 in the denosumab group. Plain radiographs of the thoraco-lumbar spine were performed in all these patients, and none showed signs of vertebral compression or deformity.

### Implant survival and heterotopic ossification

2 early stem revisions due to aseptic loosening were recorded—1 in each group—within the first 2 years after surgery. 1 case of cup revision in the treatment group due to hip joint instability was performed after 3 years.

Radiographic assessment showed no signs of loosening around the implants.

Of all investigated participants, 65% had heterotopic ossification with Brooker classification 1 or more at the 60-month follow-up (Table 4). There was no statistically significant difference in the proportion of participants who developed heterotopic ossification between groups.

**Table 4:** Implant revision and heterotopic ossification rates at medium-term follow-up. Values are count (%)

Factor	Total n = 54	Denosumab n = 27	Placebo n = 27	P value
Heterotopic ossification				
0	19	10	9	0.6
1	23	12	11	
2	11	4	7	
4	1	1	0	
> 1	35 (65)	17 (63)	18 (67)	
Revision	3 (5.6)	2 (7.4)	1 (3.7)	0.5

## Discussion

This is among the largest cohorts to longitudinally evaluate denosumab in a non-osteoporotic THA population under controlled conditions.

We aimed to assess the medium-term effects of denosumab on pBMD. Approximately 5 years following uncemented THA, early treatment with 2 doses of denosumab did not result in statistically significant differences in pBMD or clinical outcomes compared with placebo. Although previous analyses showed pBMD preservation at 12 and 24 months, this benefit was not sustained after 5 years. Denosumab effectively prevented early periprosthetic bone loss during the first year after THA. Following treatment cessation, pBMD gradually declined and approached the levels of the control group between 2 and 5 years, without evidence of accelerated bone loss or related adverse clinical events. No vertebral fractures or serious adverse skeletal events were observed during the medium-term follow-up in this non-osteoporotic cohort. No difference was seen in the BMD level of the unaffected hip or vertebrae L1–L4.

### Interpretation and comparison with previous studies

Our findings are consistent with previous randomized trials demonstrating that denosumab effectively attenuates early periprosthetic bone loss after uncemented THA [7,9]. Importantly, the observed long-term decline in pBMD after treatment cessation is not identical to the phenomenon termed “rebound,” a process during which BMD declines to below pre-treatment levels after cessation of denosumab. This phenomenon has been linked to increased fracture risk, especially in individuals with underlying skeletal fragility [16]. While this complication is now well recognized, it was not widely acknowledged when this study was initiated.

The European Calcified Tissue Society (ECTS) has issued a position statement recommending subsequent bisphosphonate therapy to mitigate the rebound risk following denosumab discontinuation [17]. This strategy has shown success in postmenopausal osteoporosis, yet its efficacy in arthroplasty patients is unknown. Recent work by Martín-Pérez et al. and others underscores the increased fracture risk and suggests the need for carefully planned sequential antiresorptive therapy when discontinuing denosumab [18,19]. Interestingly, the underlying mechanism may involve reactivation of osteoclast precursors, including osteomorphs, as proposed by Kim et al. [20].

The absence of clinically relevant rebound effects in the present cohort should be interpreted in light of the specific biological and clinical context. Clinically significant rebound after denosumab discontinuation has predominantly been described in skeletally fragile populations exposed to prolonged RANKL inhibition, where rapid post-cessation increases in bone turnover coincide with pre-existing microarchitectural vulnerability and translate into an excess risk of vertebral fractures. In contrast, the patients in this study were non-osteoporotic, received only short-term denosumab exposure, and were followed in a predominantly local, periprosthetic bone compartment. Under these conditions, treatment cessation was associated with a gradual convergence of pBMD toward control levels rather than an overshoot below baseline, and without accompanying vertebral fractures or other adverse skeletal events. These findings suggest that concerns related to denosumab rebound, while highly relevant in osteoporosis management, may not directly apply to short-course perioperative use in low-risk arthroplasty patients and should not be extrapolated uncritically across fundamentally different clinical settings [24].

Although none of the participants in our study had osteoporosis by WHO definition (T-score < –2.5), a rebound phenomenon was a potential outcome in our cohort. However, this was not observed; we rather found a gradual decline in pBMD in the treatment group. All participants were systematically evaluated for back pain and underwent spinal radiographs when indicated. No vertebral fractures were found, supporting the relative safety of short-course denosumab in this patient group, although our study was not powered to detect rare events, and lumbar radiographs were obtained only in symptomatic participants during our medium-term follow-up.

No statistically significant differences between the denosumab and placebo groups were observed at 5 years for either lumbar spine or contralateral hip BMD. These findings suggest that denosumab discontinuation was not associated with an excess long-term decline in BMD at skeletal sites not exposed to surgery in this study population.

Regarding surgical outcomes, these are consistent with national registry data on the cumulative revision rate of the implants used [25]. While denosumab has been hypothesized to reduce early loosening by preserving proximal bone stock, our data does not support this effect in non-osteoporotic patients. Notably, a large registry study in Denmark found that long-term bisphosphonate use in osteoporotic patients reduced revision risk, whereas short-term use increased infection-related revisions [26]. Similar registry-based studies for denosumab are lacking, likely due to lower usage rates and the need for multinational collaboration to detect rare adverse events.

### Heterotopic ossification

Heterotopic ossification was detected in approximately 60% of participants at the 5-year follow-up, with no significant difference between groups. This rate falls within the upper range of previous reports, which vary widely from 10% to 60% [27,28]. The direct lateral approach used in this trial has been associated with a lower heterotopic ossification prevalence (~24%) in prior literature [27], making the relatively high frequency in our cohort unexpected. We abstained from routine NSAID prophylaxis in our trial participants, for obvious reasons, and because NSAIDs are known to prevent the formation of heterotopic ossification this may present a possible explanation for the observed phenomenon [29,30].

### Strengths

The cohort was previously included in a prospective, randomized, double-blind trial with placebo control and independent funding. The blinding of both participants and investigators minimized potential performance and detection bias. The consistency in surgical technique, implant selection, and follow-up protocol provided a controlled environment for evaluating treatment effects. DEXA was used to rigorously quantify pBMD in all participants. Furthermore, the statistical approach incorporated linear mixed models to account for baseline imbalances and repeated measures over time, while adjusting for individual variability, including sex.

### Limitations

The restrictive inclusion criteria—BMI < 35, age ≤ 65, and unilateral OA—limit generalizability. These criteria reflect the prevailing clinical norms for uncemented THA in Sweden at the time, yet they may exclude patients at highest risk for pBMD loss and prosthesis failure. Consequently, the low baseline risk in our cohort may have attenuated the potential benefit of antiresorptive treatment.

Nevertheless, a study by Aro et al. [7] reported similar effects of denosumab on pBMD, despite notable differences in study design. Their cohort consisted exclusively of older female patients—on average more than a decade older than ours, a different cementless implant system was utilized, and denosumab had already been administered 1 month prior to surgery. Despite these differences, that study found that denosumab increased pBMD in the clinically relevant regions of the proximal femur, which lends support to the broader applicability of our results across different patient populations and clinical settings.

The use of pBMD as a surrogate outcome, rather than a direct clinical endpoint such as revision for loosening, introduces further uncertainty regarding clinical relevance. Although changes in pBMD are associated with prosthesis stability, the magnitude of risk reduction for revision remains undefined. Tertiary outcomes—such as heterotopic ossification and implant revision—were not part of the power calculation and may be underpowered to detect significant group differences, raising the possibility of type II error. Moreover, the study lacked radiostereometric analysis (RSA), a method that could have provided early insights into micromotion and implant stability. Systematic vertebral fracture assessment was not included in the protocol, and thoraco-lumbar radiographs were performed only in participants reporting back pain; thus, asymptomatic vertebral fractures may have gone undetected. Another potential limitation is that the study did not include a predefined post-treatment strategy. The gradual decline in pBMD after the first year likely reflects normal stress-shielding rather than a drug-related effect, and whether sequential antiresorptive therapy could modify this pattern remains unknown.

In accordance with our ethical approval, medical charts or registry data of participants who did not attend the 5-year visit could not be assessed; therefore, no analysis of the clinical outcome of participants lost to follow-up was feasible. The baseline characteristics of the patients lost to follow-up are presented in Table 5 (see Appendix).

### Conclusion

We showed that denosumab is not associated with preserved pBMD among non-osteoporotic patients after 5 years. While the positive effects of denosumab on pBMD described in the analysis of this trial’s primary outcome after 2 years were promising, we lack evidence that they are maintained over time. This questions the clinical efficacy of short-term denosumab application in the setting of THA surgery.

*In perspective*, future studies should identify patients most likely to benefit from antiresorptive therapy and further evaluate long-term efficacy and safety in arthroplasty populations.

## Notes

CS: validation, investigation, resources, writing—original draft, visualization; NPH: methodology, writing (review and editing), supervision; HM: conceptualization, methodology, validation, formal analysis (part of), investigation, resources, writing (review and editing), supervision, project administration, funding acquisition.

The authors extend their sincere gratitude to all study participants, whose commitment and willingness to contribute their time made this research possible. They thank Catharina Strömstedt, research nurse, for excellent support, and Tatevik Ghukasyan Lakic (UCR) for all the statistical analyses. Special thanks are offered to Andreas Nyström and Demostenis Kiritopoulos for their contributions to the original articles and to Lena Karlsson for her invaluable support with the BMD measuring and the DXA device.

Handling co-editor: Keijo Mäkelä

*Acta* thanks Hannu T Aro, Jonas Ranstam, and Lene Bergendal Solberg for help with peer review of this manuscript.

## Appendix

**Table 5:** Baseline characteristic for the 10 participants who did not attend visit 7. Values are mean (SD) unless otherwise is specified

Characteristic	Denosumab (n = 5)	Placebo (n = 5)
Age	56 (8)	60 (3)
Male, n	2	2
Body mass index	26.8 (3.2)	26.4 (3.6)
Harris Hip Score, median (range)	57 (28–65)	52 (33–63)
Z-score, total hip (unaffected)	–0.36 (0.53)	0.12 (0.58)
Z-score, total hip (affected)	–0.20 (0.73)	–0.34 (0.98)
Z-score, L1–L4	0.04 (0.68)	0.36 (1.00)
T-score, total hip (unaffected)	–0.94 (0.68)	–0.64 (0.40)
T-score, total hip (affected)	–0.94 (0.96)	–1.12 (0.80)
T-score, L1–L4	–0.48 (0.73)	–0.52 (1.24)

## References

[1] Cavalli L, Brandi M L. Periprosthetic bone loss: diagnostic and therapeutic approaches. F1000Res 2013; 2: 266. doi: 10.12688/f1000research.2-266.v2. 25642325 PMC4304431

[2] Sköldenberg O G, Salemyr M O, Bodén H S, Ahl T E, Adolphson P Y. The effect of weekly risedronate on periprosthetic bone resorption following total hip arthroplasty: a randomized, double-blind, placebo-controlled trial. J Bone Joint Surg Am 2011; 93: 1857-64. doi: 10.2106/JBJS.J.01646. 22012522

[3] Khatod M, Inacio M C S, Dell R M, Bini S A, Paxton E W, Namba R S. Association of bisphosphonate use and risk of revision after THA: outcomes from a US total joint replacement registry. Clin Orthop Relat Res 2015; 473: 3412-20. doi: 10.1007/s11999-015-4263-4. 25896134 PMC4586196

[4] Namba R S, Inacio M C S, Cheetham T C, Dell R M, Paxton E W, Khatod M X. Lower total knee arthroplasty revision risk associated with bisphosphonate use, even in patients with normal bone density. J Arthroplasty 2016; 31: 537-41. doi: 10.1016/j.arth.2015.09.005. 26454569

[5] Wilkinson J M, Eagleton A C, Stockley I, Peel N F A, Hamer A J, Eastell R. Effect of pamidronate on bone turnover and implant migration after total hip arthroplasty: a randomized trial. J Orthop Res 2005; 23: 1-8. doi: 10.1016/j.orthres.2004.06.004. 15607868

[6] McDonald C L, Lemme N J, Testa E J, Aaron R, Hartnett D A, Cohen E M. Bisphosphonates in total joint arthroplasty: a review of their use and complications. Arthroplasty Today 2022; 14: 133-9. doi: 10.1016/j.artd.2022.02.003. 35308048 PMC8927797

[7] Aro H T, Nazari-Farsani S, Vuopio M, Löyttyniemi E, Mattila K. Effect of denosumab on femoral periprosthetic BMD and early femoral stem subsidence in postmenopausal women undergoing cementless total hip arthroplasty. JBMR Plus 2019; 3: e10217. doi: 10.1002/jbm4.10217. 31687650 PMC6820573

[8] Aro H T. The potential use of denosumab in patients with arthroplasty. Lancet Rheumatol 2021; 3: e165-6. doi: 10.1016/S2665-9913(20)30447-1. 38279378

[9] Nyström A, Kiritopoulos D, Ullmark G, Sörensen J, Petrén-Mallmin M, Milbrink J, et al. Denosumab prevents early periprosthetic bone loss after uncemented total hip arthroplasty: results from a randomized placebo-controlled clinical trial. J Bone Miner Res 2020; 35: 239-47. doi: 10.1002/jbmr.3883. 31589776

[10] Ledin H, Good L, Aspenberg P. Denosumab reduces early migration in total knee replacement: a randomized controlled trial involving 50 patients. Acta Orthop 2017; 88: 255-8. doi: 10.1080/17453674.2017.1300746. 28287004 PMC5434591

[11] Curtis J R, Arora T, Liu Y, Lin T-C, Spangler L, Brunetti V C, et al. Comparative effectiveness of denosumab vs alendronate among postmenopausal women with osteoporosis. J Bone Miner Res 2024; 39: 826-34. doi: 10.1093/jbmr/zjae079. 38753892 PMC11301726

[12] Miller P D, Bolognese M A, Lewiecki E M, McClung M R, Ding B, Austin M, et al. Effect of denosumab on bone density and turnover in postmenopausal women with low bone mass after long-term continued, discontinued, and restarting of therapy: a randomized blinded phase 2 clinical trial. Bone 2008; 43: 222-9. doi: 10.1016/j.bone.2008.04.007. 18539106

[13] Kiritopoulos D, Nyström A, Ullmark G, Sörensen J, Petrén-Mallmin M, Milbrink J, et al. Denosumab prevents acetabular bone loss around an uncemented cup: analysis of secondary outcomes in a randomized controlled trial. Acta Orthop 2022; 93: 709-20. doi: 10.2340/17453674.2022.4537. 36069479 PMC9450252

[14] Sköld C, Kultima K, Freyhult E, Larsson A, Gordh T, Hailer N P, et al. Effects of denosumab treatment on the expression of receptor activator of nuclear kappa-B ligand (RANKL) and TNF-receptor TNFRSF9 after total hip arthroplasty-results from a randomized placebo-controlled clinical trial. Osteoporos Int 2022; 33: 1-8. doi: 10.1007/s00198-022-06423-w. PMC9463208 35608639

[15] Li X, Han J, Shi X, Bi Z, Liu J. Zoledronic acid and denosumab for periprosthetic bone mineral density loss after joint arthroplasty: a systematic review and meta-analysis of randomized controlled trials. Arch Osteoporos 2023; 18: 37. doi: 10.1007/s11657-023-01227-9. 36840811

[16] Lamy O, Gonzalez-Rodriguez E, Stoll D, Hans D, Aubry-Rozier B. Severe rebound-associated vertebral fractures after denosumab discontinuation: 9 clinical cases report. J Clin Endocrinol Metab 2017; 102: 354-8. doi: 10.1210/jc.2016-3170. 27732330

[17] Tsourdi E, Langdahl B, Cohen-Solal M, Aubry-Rozier B, Eriksen E F, Guañabens N, et al. Discontinuation of denosumab therapy for osteoporosis: a systematic review and position statement by ECTS. Bone 2017; 105: 11-17. doi: 10.1016/j.bone.2017.08.003. 28789921

[18] Martín-Pérez M, Sánchez-Delgado B, García-Poza P, López-Álvarez S, Martín-Merino E. Multiple vertebral fractures after antiosteoporotic medications discontinuation: a comparative study to evaluate the potential rebound effect of denosumab. Bone 2025; 190: 117325. doi: 10.1016/j.bone.2024.117325. 39521365

[19] Sølling A S, Harsløf T, Langdahl B. Treatment With zoledronate subsequent to denosumab in osteoporosis: a 2-year randomized study. J Bone Miner Res 2021; 36: 1245-54. doi: 10.1002/jbmr.4305. 33813753

[20] Kim A S, Girgis C M, McDonald M M. Osteoclast recycling and the rebound phenomenon following denosumab discontinuation. Curr Osteoporos Rep 2022; 20: 505-15. doi: 10.1007/s11914-022-00756-5. 36201122 PMC9718877

[21] Digas G, Kärrholm J, Thanner J. Different loss of BMD using uncemented press-fit and whole polyethylene cups fixed with cement: repeated DXA studies in 96 hips randomized to 3 types of fixation. Acta Orthop 2006; 77: 218-26. doi: 10.1080/17453670610045948. 16752282

[22] Gruen T A, McNeice G M, Amstutz H C. “Modes of failure” of cemented stem-type femoral components: a radiographic analysis of loosening. Clin Orthop Relat Res 1979; (141): 17-27. 477100

[23] Brooker A F, Bowerman J W, Robinson R A, Riley L H J. Ectopic ossification following total hip replacement: incidence and a method of classification. J Bone Joint Surg Am 1973; 55: 1629-32. PMID: 4217797 4217797

[24] Kumar S, Wang M, Kim A S, Center J R, McDonald M M, Girgis C M. Denosumab discontinuation in the clinic: implications of rebound bone turnover and emerging strategies to prevent bone loss and fractures. J Bone Miner Res 2025; 40: 1017-34. doi: 10.1093/jbmr/zjaf037. 40057981 PMC12406127

[25] SAR – Annual report 2023 Swedish Arthroplasty Register. doi: 10.18158/r1HHfziah n.d.

[26] Thillemann T M, Pedersen A B, Mehnert F, Johnsen S P, Søballe K. Postoperative use of bisphosphonates and risk of revision after primary total hip arthroplasty: a nationwide population-based study. Bone 2010; 46: 946-51. doi: 10.1016/j.bone.2010.01.377. 20102756

[27] Herzberg R, Tracey O C, Tahvilian S, Baksh N, Zikria B, Naziri Q. Incidence of heterotopic ossification following total hip arthroplasty by approach: a systematic review. Eur J Orthop Surg Traumatol 2024; 34: 2089-98. doi: 10.1007/s00590-024-03896-9. 38536499

[28] Purcell K F, Lachiewicz P F. Heterotopic ossification after modern total hip arthroplasty: predisposing factors, prophylaxis, and surgical treatment. J Am Acad Orthop Surg 2023; 31: 490-6. doi: 10.5435/JAAOS-D-22-01070. 36972521

[29] Beckmann J T, Wylie J D, Potter M Q, Maak T G, Greene T H, Aoki S K. Effect of naproxen prophylaxis on heterotopic ossification following hip arthroscopy: a double-blind randomized placebo-controlled trial. J Bone Joint Surg Am 2015; 97: 2032-7. doi: 10.2106/JBJS.N.01156. 26677237 PMC4673445

[30] Zhang A-H, Chen X, Zhao Q-X, Wang K-L. A systematic review and meta-analysis of naproxen for prevention heterotopic ossification after hip surgery. Medicine 2019; 98: e14607. doi: 10.1097/MD.0000000000014607. 30946309 PMC6455982

